# The deleterious effect of WHO grade II diffuse glioma on socioeconomic status as evaluated through occupation

**DOI:** 10.1038/s41598-025-08240-7

**Published:** 2025-07-02

**Authors:** Matthew A. Kirkman, Michael S. C. Thomas, Andrew Tolmie

**Affiliations:** 1 Department of Psychology and Human Development, UCL Institute of Education, University College London, 25 Woburn Square, London, WC1H 0AA UK; 2 Department of Psychological Sciences, Birkbeck, University of London, London, UK

**Keywords:** Cognitive outcomes, Educational attainment, Low-grade glioma, Quality of life, Socioeconomic status, CNS cancer, Prognosis, Quality of life

## Abstract

Although previous studies have evaluated return-to-work status following low-grade glioma (LGG) treatment, there is a paucity of detailed data on the nature of the occupation a patient returns to. The aim of this study was to describe the socioeconomic status of patients with WHO grade II diffuse glioma over long-term follow up and whether it changed following the LGG diagnosis. In addition, cognitive, education, and quality of life profiles, as well as estimated premorbid intelligence, were evaluated to explore the possibility of relationships with socioeconomic status. Twenty-one individuals with a histological diagnosis of WHO grade II diffuse glioma were recruited prospectively from a single UK center and completed questionnaires evaluating general health, socioeconomics, education, quality of life, and a battery of validated cognitive tests. Socioeconomic status was evaluated through an occupational-based classification. Forty percent of participants in the most advantaged socioeconomic group prior to the diagnosis of a glioma did not remain in the same advantaged group at the point of recruitment into the study. This is despite no marked cognitive impairments across the cohort and no significant cognitive differences between those who did and did not remain in this advantaged group. Attainment of a university-level qualification was significantly associated with maintenance of socioeconomic advantage following the LGG diagnosis, which may be explained by the concept of cognitive reserve. In conclusion, there are profound deleterious socioeconomic effects of LGG and its treatment. Educational attainment may be protective. Early tailored and proactive professional support regarding work-related issues is required for this patient group.

**Supplementary Information:**

The online version contains supplementary material available at 10.1038/s41598-025-08240-7.

## Introduction

Gliomas are the most frequent type of primary brain tumor in adults^1^, with an age-adjusted annual incidence of 7.3 cases per 100,000 person-years^2^. The most recent World Health Organization (WHO) classification of tumors of the central nervous system^3^ groups gliomas into several categories based on molecular and immunohistochemical characteristics, the commonest group encountered in clinical practice being adult-type diffuse gliomas, comprising isocitrate dehydrogenase (IDH)-mutant astrocytoma, IDH-mutant and 1p/19q-codeleted oligodendroglioma, and IDH-wildtype glioblastoma. This classification, alongside tumor grade, plays an important role in the treatment options offered to patients as well as their prognosis.

Diffuse WHO grade II gliomas are part of a group defined as low-grade gliomas (LGGs) and many patients with diffuse WHO grade II gliomas survive with stable disease for several years or longer, prior to dedifferentiation of their tumor into a more malignant form^4,5^. The therapies used to treat patients with gliomas and other forms of brain tumor have been associated with a number of adverse consequences, including effects on an individual’s cognition^6^ and health-related quality of life (HRQOL)^7,8^, and cognitive function and HRQOL have been shown to be closely linked even in patients with stable LGG^9^. The optimization of HRQOL—particularly during the stable phase of the disease—is of crucial importance in this patient group. Their HRQOL is likely to be influenced by a range of factors in addition to the underlying tumor and treatment(s) administered, including the psychosocial consequences resulting from potential loss of employment and altered social roles^10^.

Socioeconomic status is associated with HRQOL in children^11^ and adults^12^ with chronic diseases, and across the lifespan in the general population^13,14^. A number of studies have shown the devastating effects of a brain tumor diagnosis in childhood on subsequent quality of life, cognitive function, socioeconomic status, and educational attainment^15,16^. However, the impact of an LGG diagnosis and its treatment in adulthood on socioeconomic—and particularly occupational—status have been relatively understudied. Although previous studies have evaluated return-to-work status following treatment of brain tumors including LGG^17^, there is a paucity of data on the nature of the occupation a patient returns to and whether the work is of a similar nature and status (and inherently, remuneration) or not. This is perhaps of particular importance for patients within healthcare systems such as that of the United States, where part of the direct medical costs are paid directly by patients and the economic burden of a LGG diagnosis can be substantial^18,19^.

The aim of this study was to describe the socioeconomic status of patients with WHO grade II diffuse glioma over long-term follow up and whether it changed following the LGG diagnosis. In addition, cognitive, education, and quality of life profiles, as well as estimated premorbid intelligence, were evaluated to explore the possibility of relationships with socioeconomic status. ‘Socioeconomic factors’ for the purpose of the current study comprised various social and economic factors including occupation, parental education, and level of deprivation in the area of upbringing. The current study was not designed as an epidemiological study but instead was part of a deep phenotyping project^20^.

## Methods

### Ethical approval

This study was approved by the UK National Health Service Health Research Authority (project approval ID: 233329). All participants provided written informed consent prior to participation in the study.

### Study design and setting

Data were prospectively collected through a self-designed questionnaire combined with validated measures/tools to ascertain information on education, socioeconomic factors, tobacco/alcohol consumption, medical and medication/drug history, cognitive status, quality of life, personality type, and premorbid IQ. These were administered to participants by the first author of this manuscript (MAK) at the National Hospital for Neurology and Neurosurgery (NHNN), Queen Square, London, UK.

### Participants

We contacted, by postal mail, all individuals with a histological diagnosis of WHO grade I or II glioma confirmed at NHNN between 1st January 2006 and 31st December 2017 inclusive, who were aged 16 years or older at the time of diagnosis. Individuals within three months of surgery or active oncological treatment, those not understanding written or spoken English, and those lost to clinical follow-up were excluded.

### Variables and outcome measures

All participants completed the following questionnaires and assessments in the same order.

#### General questionnaire

A general questionnaire designed by the study authors collected data on age, handedness (self-reported), ethnicity (based on the classification used in the 2011 UK Government Census^21^), sex, performance (functional) status (using the Eastern Cooperative Oncology Group [ECOG] scale^22^), highest level of education prior to brain tumor diagnosis, education completed following brain tumor diagnosis, type of school attended (private/state/outside the UK/unknown), recipient of free school meals, parental/legal guardian occupation(s), parental/legal guardian degree status, household income support status, postcode of the main house the participant was raised in, participant’s occupation prior to brain tumor diagnosis, participant’s current occupation, treatment(s) received at other hospitals, other medical history/comorbidities, medication and recreational drug, alcohol, and smoking history.

#### Indices of multiple deprivation

Indices of Multiple Deprivation (IMD) data were used to facilitate socioeconomic classification. The IMD is the official measure of relative deprivation in England and defines deprivation through multiple measures including income, employment, education, health, crime, barriers to housing and services, and living environment^23^. IMD data provide a *relative* rank of each small area (average population = 1500) in England from the most to least deprived, which was used in combination with the home postcodes of participants who spent most of their childhood in England. Further information about the IMD and the different associated measures is provided in the Supplementary Methods. For the purposes of the current analyses, decile IMD data were used for relative comparisons given the small numbers of participants.

#### Occupational status

Occupational data on participants and their parents/legal guardians were collected through the general questionnaire and analyzed using the UK National Statistics Socio-economic Classification (NS-SEC) and Standard Occupational Classification 2020^24^. The analytic class of each participant’s occupation, both before and after the LGG diagnosis, and that of their parents/legal guardians were determined using tables available from the Office for National Statistics website^25^. In the current study, occupation following the LGG diagnosis was defined as the occupation of the participant at the time of study participation. The three-class version of the NS-SEC was used to group participants and their parents/legal guardians into one of the following categories: (1) higher managerial, administrative, and professional occupations; (2) intermediate occupations; and (3) routine and manual occupations. The three-class version of the NS-SEC was used instead of the five- and eight-class versions because (1) the use of a more granular classification system in the context of a small number of study participants would have further hindered the performance of meaningful statistical analyses, and (2) the more ordinal nature of the three-class version of the NS-SEC—resulting from the exclusion of a separate class of self-employed individuals in this version—facilitated statistical analyses.

#### Cognitive assessment

A battery of cognitive tests were administered to participants, including the Mini-Mental State Examination (MMSE)^26^ as a global measure of cognition, the Hopkins Verbal Learning Test-Revised (HVLT-R)^27^ as an assessment of memory, the Multilingual Aphasia Examination Controlled Oral Word Association Test (COWAT)^28^ as an assessment of verbal fluency, part A of the Trail Making Test (TMT)^29^ as an assessment of visual-motor scanning speed and visual speed working memory, part B of the TMT^29^ as a measure of executive function and visual speed working memory, the Stroop Test—Victoria version^30^ as a test of inhibitory control, and the Hayling and Brixton Tests^31^ as tests of response initiation/suppression and of visuospatial sequencing/rule attainment, respectively.

The nature of the tests and the scoring techniques applied are described in greater detail in the Supplementary Methods, including Supplementary Table S1. This set of tests was chosen to allow a comprehensive assessment of cognition, incorporating a battery recommended by the Response Assessment in Neuro-oncology (RANO) working group^32^ (MMSE, HVLT-R, COWAT, and TMT parts A and B) as well as additional tests (Stroop Test—Victoria version and the Hayling and Brixton Tests).

#### Quality of life

Participants were administered the European Organisation for Research and Treatment of Cancer (EORTC) Quality of Life Questionnaires (QLQ)-C30 and -BN20 and the Functional Assessment of Cancer Therapy—Brain (FACT-Br).

The EORTC QLQ-C30 is a 30-item instrument designed to measure quality of life in patients with cancer. It is the main ‘core’ module of the quality-of-life questionnaires devised by the EORTC, and asks responds about their experiences across a range of potential issues faced by patients with cancer, such as mobility, self-care, symptoms, and effects on family life, social activities, and finances. Version 3.0 of the questionnaire was used in the current study. The EORTC QLQ-BN20 is an additional module that was administered to participants, and focuses on the quality of life of patients with brain tumors. It consists of 20 items that assess a range of potential concerns and symptoms experienced by patients with brain tumors, including future uncertainty as well as visual, mobility, and communication difficulties. Further information about the EORTC QLQ-C30 and QLQ-BN20 is provided in the Supplementary Methods, including Supplementary Tables S2 and S3.

The FACT-Br is a 50-item instrument that evaluates general well-being and brain cancer-specific concerns, with the items grouped into the following categories: physical well-being, social/family well-being, emotional well-being, functional well-being, and additional concerns, with the initial four categories representing general items and the latter being used to generate a brain cancer subscale. Further information is provided in the Supplementary Methods, including Supplementary Table S4.

#### Estimated premorbid intelligence

The NART^33^ is a validated tool that estimates premorbid intelligence through measuring crystallized intelligence stored in knowledge and skills such as vocabulary and pronunciation, and was used in the current study. The NART is a single-word, oral reading test that comprises of 50 words that are all irregular in that they violate grapheme-phoneme correspondence rules (an example is the word ‘chord’). As the words involved are irregular, intelligent guessing should not provide the correct answer, and instead the test is a measure of previous word knowledge. Furthermore, as the test utilizes only single words, the stimulus that a participant needs to analyze is not complex, thus minimal demands on current cognitive capacity are made. The test originated from the finding that, in patients with dementia, oral reading is often preserved despite reading for meaning being commonly impaired.

The number of incorrectly pronounced words were summated to provide a ‘total NART errors’ score, in line with standard scoring procedures^33^, and this score was incorporated into a linear regression equation to predict scores in the Wechsler Adult Intelligence Scale version IV (WAIS-IV) following a previously published and validated technique based on a British sample^34^, as follows:

$${\text{Predicted WAIS-IV Full Scale IQ}} = - 0.{975 } \times {\text{ NART}}\;{\text{error}}\;{\text{score}} + {126}.{41}$$

#### Clinical data

Electronic medical records (Epic software; Epic Systems Corporation, Verona, WI, USA) were reviewed to triangulate data provided in the general questionnaire and to obtain detailed clinical information, including: date of diagnosis; clinical symptoms at the time of diagnosis; confirmation of the specific tumor type diagnosed, including the WHO grade (using dedicated and detailed histopathology reports, where available); other histopathological and molecular or genetic details regarding the tumor; treatment(s) received for the brain tumor, including the number of operations performed and the administration of chemotherapy and/or radiotherapy, as well as the chronology of the treatment(s) and the time since the last surgery/chemotherapy/radiotherapy treatment; the use of anti-epileptic medication(s), including the number, type, and dose of medication(s) used; complication(s) associated with either the brain tumor itself or the treatment(s) administered; other medical conditions and their potential impact on the brain tumor and its management; other medication(s) taken by the participant that were not directly related to the management of the brain tumor; and clinical status of the participant during the follow-up period, including information regarding tumor recurrence, if relevant.

### Statistical analysis

Summary data are presented as mean (standard deviation) and median (range) for completeness. Shapiro–Wilk tests were calculated to evaluate normality and, for consistency, parametric tests have been reported, although those data not normally distributed also underwent testing with equivalent non-parametric tests to ensure the use of parametric statistical tests did not significantly alter the interpretation of any of the results reported.

Comparison of participants’ NS-SEC class prior to and following the LGG diagnosis was calculated using a 2 × 2 McNemar’s chi-squared test. Furthermore, for comparison of the distribution of NS-SEC classes within the study population to that of London, a 2 × 2 standard chi-squared test was calculated. To evaluate whether changes in socioeconomic status following the LGG diagnosis were associated with potentially relevant variables collected during the study, *t*-tests (for continuous variables) and chi-squared tests (for categorical variables) were calculated by comparing participants who remained in the most advantaged NS-SEC Class group (Class 1) following the LGG diagnosis compared to those in the remaining groups according to demographic (sex, age), socioeconomic (IMD decile), educational (years of schooling, educational attainment of participant [degree vs no degree], educational attainment of parents [at least one parent with a degree vs none with a degree]), and clinical variables (tumor location [frontal vs non-frontal], tumor laterality [right vs left], number of surgeries for the brain tumor [1 vs 2], extent of tumor resection [gross total resection vs not gross total resection], received chemotherapy [yes vs no], received radiotherapy [yes vs no], anti-epileptic drug administration [yes vs no], 1p19q codeletion status [codeleted vs not], performance status, time since diagnosis, time since last surgery, time since last chemotherapy, and time since last radiotherapy), as well as estimated premorbid intelligence (NART-predicted premorbid IQ).

All statistical analyses reported in the study were performed using SPSS version 25 (IBM Corporation, Armonk, NY, USA). A P-value statistical significance threshold of 0.05 was applied. This study was considered exploratory in nature and correction for multiple comparisons was not performed. Although this increases the risk of a type I statistical error, correction techniques are often too strict, reducing statistical power and increasing the risk of missing potentially important findings through a type II statistical error. Some advocate against the use of corrections for multiple testing completely^35^, and literature supports this approach for exploratory studies such as this^36^.

## Results

### Participants

**Fig. 1:**
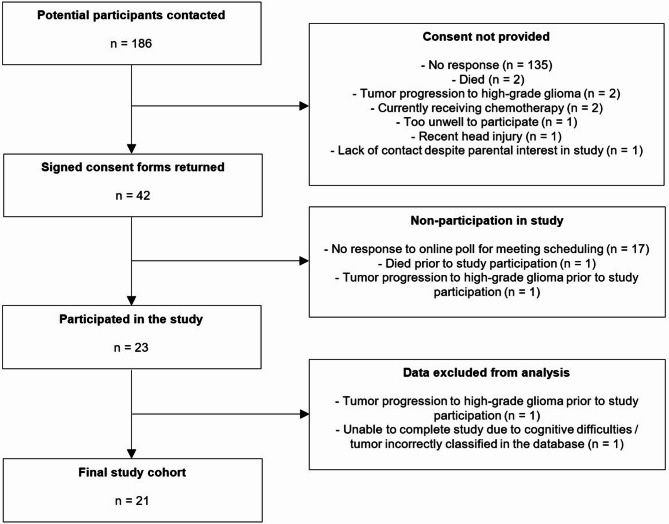
Study flowchart.

In total, 186 potential participants were identified as being potentially eligible to participate in the study and were contacted by postal mail. Of those who did not respond within two weeks, a reminder was sent by email (where available). Forty-two potential participants responded to indicate their agreement, and 21 of these were included in the final study. A flow chart demonstrating the recruitment steps is shown in Fig. 1.

### Patient characteristics

A summary of the participants’ demographics is provided in Table 1, and detailed information regarding performance (functional status), medical and drug history, treatments received in relation to the LGG, and tumor as well as other clinical characteristics are provided in the Supplementary Results, including Supplementary Tables S5–S8. The mean age of study participants was 47 (standard deviation [SD] 9.6) years (male:female 11:10). The majority of individuals (15 of 21; 71.4%) self-reported as White British. The participants were a mean of 57.5 (SD 24.8) months following histopathological confirmation of their brain tumor at the time of study participation. The majority of participants (n = 16; 76.2%) self-reported as ECOG grade 0, i.e., fully active and able to carry on all pre-disease performance without restriction. Most (n = 16; 76.2%) were also receiving anti-epileptic drugs at the time of study participation. While most participants (n = 18; 85.7%) consumed alcohol with a variable frequency, only one (4.8%) participant claimed to be a current tobacco smoker.

**Table 1:** Demographics of the study population.

Variable	N (%) or mean (SD)/median (range)
Age at study participation in years	47 (9.6)/46 (31–72)
Sex, M:F	11:10 (52.4%:47.6%)
Hand dominance, right:left	20:1 (95.2%:4.8%)
Ethnicity	
White British, English, Northern Irish, Scottish or Welsh	15 (71.4%)
Any other white background	2 (9.5%)
Indian	2 (9.5%)
Any other Asian background	1 (4.8%)
Caribbean	1 (4.8%)
Time between histological diagnosis and study participation in months	57.5 (24.8)/52.8 (22.5–106.4)

SD, standard deviation.

The majority (n = 14; 66.7%) of patients were diagnosed as having a WHO grade II oligodendroglioma, and the remainder of patients were diagnosed as having a WHO grade II diffuse astrocytoma (n = 6; 28.6%) or WHO grade II oligoastrocytoma (n = 1; 4.8%). The patient diagnosed with a WHO grade II oligoastrocytoma would likely be diagnosed with a WHO grade II oligodendroglioma using current diagnostic criteria (particularly given the confirmed presence of a 1p19q codeletion; information about 1p19q, IDH, and ATRX status are provided in the Supplementary Results, including Supplementary Table S6). The majority of tumors were right-sided (n = 13; 61.9%) and the frontal lobe was the most commonly affected lobe (n = 13; 61.9%). In the majority of participants (n = 15; 71.4%), the initial symptom or sign at the time of presentation to healthcare providers was seizures.

All participants underwent surgery to confirm the histopathological diagnosis. Most (n = 15; 71.4%) participants underwent one surgical procedure, but six (28.6%) underwent two. Six (28.6%) and 8 (38.1%) participants received chemotherapy and radiotherapy for their brain tumor, respectively.

### Educational attainment

Study participants completed a median of 15.5 (range = 9–24) years of education prior to study participation. The majority of participants were educated to degree level (Professional qualification (n = 2, 9.5%; Bachelor’s degree: n = 10; 47.6%; Master’s degree: n = 1; 4.8%) prior to the diagnosis of a glioma. Following the diagnosis, one (4.8%) participant completed a Master’s degree and four (19.0%) completed diplomas or vocational qualifications.

### Socioeconomic status

The socioeconomic information provided by participants, including parental occupational and educational status, is summarized in the Supplementary Results, including Supplementary Tables S9–S12.

The occupations of participants before and following the glioma diagnosis are shown in Table 2, where each row represents the same participant’s occupation before and following the diagnosis. Over half (n = 12; 57.1%) of participants in the study changed their occupation following the glioma diagnosis, 8 (38.1%) did not change their occupation, and 1 (4.5%) maintained the same occupation but reduced their working hours. One (4.5%) participant who changed their occupation (from solicitor to medical ethics and law work) also reduced their working hours.

**Table 2:** Participant occupations and associated NS-SEC class prior to and following the LGG diagnosis.

Occupation prior to glioma diagnosis	Occupation following glioma diagnosis
Airline pilot (NS-SEC Class 1)	Handyman and car ferrier (NS-SEC Class 3)
Creative director (NS-SEC Class 1)	Creative director (NS-SEC Class 1)
Personal assistant (NS-SEC Class 2)	Executive assistant and office coordinator (NS-SEC Class 2)
Project manager (NS-SEC Class 1)	Project manager (NS-SEC Class 1)
Solicitor (NS-SEC Class 1)	Medical ethics and law work (part-time) (NS-SEC Class 1)
Machine setter (NS-SEC Class 3)	Machine setter (now part-time) (NS-SEC Class 3)
Civil servant (NS-SEC Class 2)	Civil servant (NS-SEC Class 2)
Accountant/lecturer (NS-SEC Class 1)	Unemployed (−)
Finance manager (NS-SEC Class 1)	Finance administrator (NS-SEC Class 2)
Internal communications officer (NS-SEC Class 3)	Unemployed (−)
Team leader (NS-SEC Class 3)	Warehouse operative (NS-SEC Class 3)
Public health information analyst (NS-SEC Class 1)	Public health information analyst (NS-SEC Class 1)
IT consultant (NS-SEC Class 1)	IT consultant (NS-SEC Class 1)
Sports massage therapist (NS-SEC Class 1)	Unemployed (−)
Company director/Engineering consultant (NS-SEC Class 1)	Company director/Engineering consultant (NS-SEC Class 1)
Project analyst (NS-SEC Class 1)	Project analyst (NS-SEC Class 1)
Careers advisor (NS-SEC Class 1)	Trainee education mental health practitioner (NS-SEC Class 2)
Teacher (NS-SEC Class 1)	Teacher (NS-SEC Class 1)
Diplomat (NS-SEC Class 2)	Unemployed (−)
Planning manager (NS-SEC Class 1)	Planning manager (NS-SEC Class 1)
Computer IT helpdesk analyst (NS-SEC Class 1)	Permitting officer—highways (NS-SEC Class 2)

NS-SEC classes are shown in parentheses.

NS-SEC, National Statistics Socio-economic Classification.

**Fig. 2:**
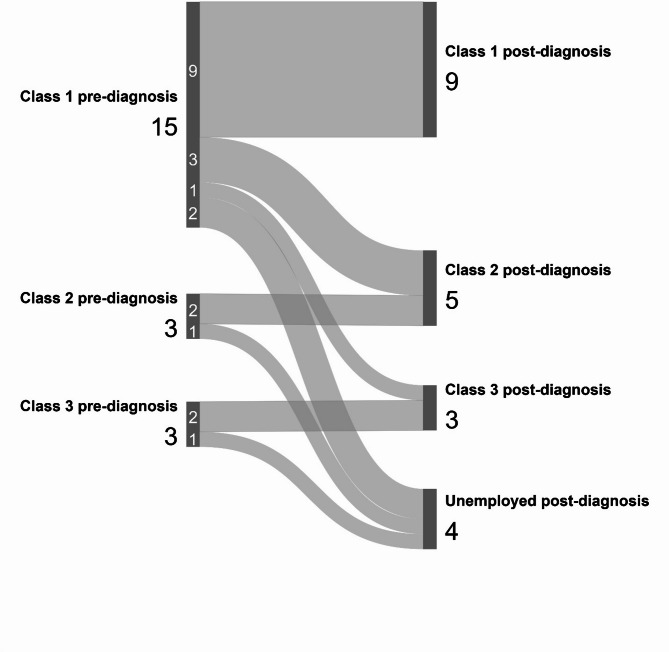
Sankey plot demonstrating National Statistics Socio-economic Classification data from the study participants prior to and following low-grade glioma diagnosis.

The NS-SEC classifications for participants in the current study prior to and following the glioma diagnosis are shown in Fig. 2; Class 1 represents the most advantaged and Class 3 the least advantaged group. It is notable that the vast majority of participants (n = 15 of 21, 71.4%) were in NS-SEC Class 1 prior to being diagnosed with a brain tumor, and 40% of these (n = 6) did not maintain their NS-SEC Class 1 status through to the point of participation in the study. None of the participants were unemployed prior to the brain tumor diagnosis; however, four (19.0%) participants were unemployed at the time of study participation. The pre-/post-diagnosis change in participants’ NS-SEC class was statistically significant (2 × 2 McNemar’s chi-squared test; *p* = 0.031).

The proportion of individuals in the most advantaged NS-SEC group (Class 1) prior to their LGG diagnosis was significantly higher than expected based on the population of London evaluated through 2011 Census data ^37^ (chi-square value = 11.843, 1 df, *p* < 0.001; further detail is provided in the Supplementary Results, including Supplementary Table S12).

### Indices of multiple deprivation

Fourteen (66.7%) participants provided full postcode data from their childhood household. Of the remaining participants, one (4.8%) lived abroad during their childhood and the remainder provided only partial postcodes, insufficient for analysis using IMD data.

As can be seen from Supplementary Table S13, half (n = 7; 50%) of the 14 participants who provided full postcode data from their childhood household grew up in the 30% least deprived neighborhoods in England according to the overall IMD score. No participants grew up in the 10% most deprived neighborhoods in England.

### Cognitive assessment

Summary cognitive testing data are presented in Table 3 (more detail is provided in Supplementary Table S14). Overall performance across all cognitive tests was largely in line with normative data where available, except for scores in the HVLT-R where performance was relatively lower, and scores in the COWAT where performance was slightly higher than average.

**Table 3:** Cognitive test results.

Test name	Scores, N (%) or mean (SD)/median (range)	Normative values (where available) and interpretation of participant data (in bold)
MMSE	29.0 (1.1)/29 (27–30)	≥ 24 or 25/30 widely considered within normal limits (Salis et al., 2021) All participants within normal limits
HVLT-R	HVLT-R Overall T score: 300.1 (56.1)/290 (166–398)	The overall T score had a possible range of ≤ 140 through to ≥ 560 Participants had low-to-average performance
COWAT	43.2 (11.1)/42 (21–62) (post-adjustment scores)	Mean COWAT score of 40.1 (SD = 10.5) in age- and education-matched normative data (Ruff et al., 1996) Participants had a slightly higher overall performance compared to the normative sample
TMT	Combined A + B: 118.9 s (52.6)/99 s (74–196 s)	Normative 50th percentile (i.e., median) scores for Part A range from 23 s for the 25–34 years old group up to 38 s for the 70–74 years old group; for Part B, the equivalent 50th percentile scores range from 50 s for the 25–34 years old group to 97 s for the 70–74 years old group (Tombaugh, 2004) At a high level, participant median Part A and B TMT scores were not markedly deranged from the normative samples
Stroop Test (Victoria version)	Stroop interference ratio 1.9 (0.4)/1.9 (1.4–2.9)	Normative data from a cohort of 272 healthy, community-dwelling adults aged 18–94 years (mean education 13 years, 64% females) living in Canada: mean (SD) of 2.0 (0.6) in the 18–39 year olds to 2.7 (1.0) in the 70–79 year olds (Troyer et al., 2006) Overall participant performance at least in keeping with normative averages
Hayling Sentence Completion Test	Hayling Overall Scaled Score, n (%): 10 (very superior) = 0 9 (superior) = 0 8 (good) = 1 (4.8%) 7 (high average) = 1 (4.8%) 6 (average) = 16 (76.2%) 5 (moderate average) = 2 (9.5%) 4 (low average) = 1 (4.8%) 3 (poor) = 0 2 (abnormal) = 0 1 (impaired) = 0	As indicated by the key words accompanying scores (see left), the majority of participants had “average” performance
Brixton Spatial Anticipation Test	Brixton Scaled Score, n (%): 10 (very superior) = 1 (4.8%) 9 (superior) = 0 8 (good) = 1 (4.8%) 7 (high average) = 5 (23.8%) 6 (average) = 7 (33.3%) 5 (moderate average) = 2 (9.5%) 4 (low average) = 1 (4.8%) 3 (poor) = 1 (4.8%) 2 (abnormal) = 2 (9.5%) 1 (impaired) = 1 (4.8%)	As indicated by the key words accompanying scores (see left), the majority of participants had “average” performance

More detailed information, including the domains evaluated by each test, the full range of outcome measures (metrics) generated for each test, and the full set of scores obtained, is provided in Supplementary Table S14.

COWAT, Controlled Oral Word Association Test; HVLT-R, Hopkins Verbal Learning Test—Revised; MMSE, Mini-Mental State Examination; SD, standard deviation; TMT-A, Trail Making Test Part A; TMT-B, Trail Making Test Part B.

### Quality of life

The summary data from the quality-of-life measures are presented in Table 4. The EORTC QLQ-C30-derived global health status of participants was calculated as a median of 83.3 out of 100, with a wide range of values (25–100). The functional scales with the highest scores (indicating better performance) were physical and role functioning (both with median scores of 100/100), followed by social functioning (83.3/100) and emotional functioning (75/100). The most adversely affected function was reported to be cognition (66.7/100). Regarding the symptom scales/items, the median score for all but two of the scales/items was 0, indicating an absence of the corresponding symptom; of the remaining scales/items, the median score for insomnia was 33.3 (range 0–100) and for fatigue was 22 (0–100).

**Table 4:** Quality of life scores.

Test name	Scores, mean (SD)/median (range)
EORTC QLQ-C30 and BN20^a^	QLQ-C30 Global Health Status: 75.8 (20.2)/83.3 (25–100) Physical Functioning: 90.5 (15.3)/100 (46.7–100) Role Functioning: 77.0 (30.9)/100 (16.7–100) Emotional Functioning: 71.4 (26.8)/75 (16.7–100) Cognitive Functioning: 68.3 (22.3)/66.7 (0–100) Social Functioning: 73.8 (23.9)/83.3 (16.7–100) Fatigue: 35.4 (28.9)/22 (0–100) Nausea and Vomiting: 4.8 (9.3)/0 (0–33.3) Pain: 15.1 (30.2)/0 (0–100) Dyspnoea: 7.9 (18.0)/0 (0–66.7) Insomnia: 44.4 (37.0)/33.3 (0–100) Appetite Loss: 9.5 (23.9)/0 (0–100) Constipation: 11.1 (24.3)/0 (0–100) Diarrhoea: 12.7 (22.3)/0 (0–66.7) Financial Difficulties: 19.0 (27.0)/0 (0–66.7) BN-20 Future Uncertainty: 19.2 (23.4)/8.33 (0–83.3) Visual Disorder: 10.0 (16.9)/0 (0–55.6) Motor Dysfunction: 17.8 (27.5)/0 (0–100) Communication Deficit: 25.0 (26.2)/22.2 (0–100) Headaches: 20.0 (33.2)/0 (0–100) Seizures: 5.0 (22.4)/0 (0–100) Drowsiness: 23.3 (24.4)/33.3 (0–100) Hair Loss: 15.0 (29.6)/0 (0–100) Itchy Skin: 10.0 (21.9)/0 (0–66.7) Weakness of Legs: 8.3 (23.9)/0 (0–100) Bladder Control: 1.7 (7.5)/0 (0–33.3)
FACT-Br^b^	Physical well-being score (/28): 22.9 (3.7)/24 (14–28) Social well-being score (/28): 22.4 (4.7)/23.3 (12–28) Emotional well-being score (/24): 17.4 (4.0)/19 (7–24) Functional well-being score (/28): 21.1 (4.8)/21 (11–28) BrCS score (/92): 66.7 (15.8)/69 (32–88) FACT-Br Trial Outcome Index (/148): 110.7 (22.7)/115 (57–143) FACT-G total score (/108): 83.7 (13.7)/83 (49–107) FACT-Br total score (/200): 150.4 (27.5)/152 (81–195)

^a^In line with the standard scoring method, raw scores were standardised using a linear transformation so that scores range from 0–100.

^b^FACT-Br Trial Outcome Index is generated from the sum of the physical well-being, functional well-being, and BrCS scores; a FACT-G (general) total score is generated from the sum of the physical, social, emotional, and functional well-being scores; and a FACT-Br (brain) total score is generated from the sum of the physical, social, emotional, functional well-being, and BrCS scores.

EORTC QLQ, European Organisation for Research and Treatment of Cancer Quality of Life Questionnaires; SD, standard deviation.

The EORTC QLQ-BN20 comprised only of symptom scales/items, where a high score represents a high level of symptomatology/problems. The results indicated that the highest level of symptomatology was observed for drowsiness (median 33.3/100), followed by communication deficit (22.2/100) and future uncertainty (8.33/100). Median scores for the remaining symptom scales/items were 0.

The FACT-Br comprises of a number of subscales that can be used to generate a general (FACT-G) total score as well as a brain tumor-specific (FACT-Br) total score. Normative data^38^ from the general population (1075 US adults aged 18–91 years) and from cancer patients (2236 patients aged 18–92 years with a range of cancer sites) for the FACT-G questionnaire are shown alongside the study results in Supplementary Table S15, and more information regarding the recruitment sources of these normative data are provided in the accompanying footnote. Overall, most domain scores in the current study were similar or better to the general population and cancer patient normative data, except emotional well-being, which was lower than that in the general population (but not that in the cancer patient) normative data.

### Estimated premorbid intelligence

The median NART Total Errors Score was 16 out of a possible maximum of 50 (range 2–29), corresponding to a predicted WAIS-IV Full Scale IQ of 110.8 (98.1–124.5). Based on commonly used thresholds for WAIS-IV IQ (≥ 130, very superior; 120–129, superior; 110–119, high average; 90–109, average; 80–89, low average; 70–79, borderline; ≤ 69, extremely low^39^), all study participants had a predicted premorbid intelligence of ‘average’ or higher, with some classified as ‘high average’ or ‘superior’.

### Associations with maintenance of socioeconomic advantage

Participants in the NS-SEC Class 1 (i.e., more advantaged) group post-LGG diagnosis had, compared to the participants in the other NS-SEC groups, a significantly higher EORTC Role Functioning score (mean 92.6 [16.9] vs 65.3 [34.4], *t* = − 2.182, *p* = 0.042, 95% confidence interval = − 53.519, − 1.110), FACT Brain Cancer subscale score (mean 75.3 [9.75] vs 60.25 [16.7], *t* = − 15.053, *p* = 0.026, 95% confidence interval = − 28.135, − 1.971), and FACT-Br Trial Outcome Index score (mean 122.6 [15.3] vs 101.7 [23.7], *t* = − 2.308, *p* = 0.032, 95% confidence interval = − 39.984, − 1.956), and significantly lower EORTC Insomnia score (mean 25.9 [27.8] vs 58.3 [37.9], *t* = 2.159, *p* = 0.044, 95% confidence interval = 0.997,63.817). Participants in the NS-SEC Class 1 group post-LGG diagnosis were significantly more likely to have a university-level qualification (university level qualification = 8/9 in the NS-SEC Class 1 post-LGG diagnosis group vs 5/12 in the other NS-SEC class groups; chi-squared value = 4.863, *p* = 0.027).

There was a trend to extent of resection status being associated with NS-SEC Class 1 following LGG diagnosis, such that gross total resection was more likely in participants who were in the NS-SEC Class 1 group following diagnosis (gross total resection = 6/9 in the NS-SEC Class 1 post-LGG diagnosis group vs 3/12 in the other NS-SEC class groups; chi-squared value = 3.646, *p* = 0.056).

None of the other associations evaluated were significant, although in almost instances the HRQOL measures favored (i.e., were higher or lower depending on the specific variable under assessment) the group of participants that remained in NS-SEC Class 1 group following the LGG diagnosis.

## Discussion

This study described clinical, educational, socioeconomic, cognitive, and quality of life factors, as well as estimated premorbid intelligence, in 21 patients with histopathologically confirmed WHO grade II diffuse glioma evaluated a mean of 57.5 (SD 24.8) months following diagnosis. The demographic characteristics (age, gender) of the study population were similar to those of a national population-based study of glioma in Denmark, that found the mean age of participants with WHO grade II gliomas to be 46 years, and a slight male preponderance (54%)^2^; this indicates the current study population is representative of the wider LGG population, despite the small sample size.

There were a number of notable findings in the current study. First, over half (52.4%) of participants were educated to degree level, indicative of a population with a high overall educational attainment. Second, NS-SEC and IMD data indicate the study population were, overall, socioeconomically advantaged both in their upbringing and based on their occupations prior to the LGG diagnosis. Third, the cognitive test results demonstrated, at the group level, at least average performance compared to healthy controls in many of the tests. Fourth, all participants had an estimated average to superior WAIS-IV IQ (range: 98.1–124.5), which may have offset some of the tumor-related effects on cognition (cognitive reserve) and explain the third point, above. Fifth, the quality-of-life descriptive data indicated most results were similar to those seen in the general population and the wider group of patients with cancer^38^, with the exception of emotional well-being and cognitive functioning, which were reported as the most affected of all the domains evaluated.

Perhaps the most notable finding of all, however, was that a significant proportion of participants (40%) in NS-SEC Class 1 prior to the diagnosis of a glioma did not remain in the same socioeconomic class at the point of recruitment into the study, suggestive of the effect of the brain tumor on that participant’s life. This is despite an absence of marked impairments in all of the tested cognitive domains, and no significant differences in the cognitive performance between those who did and did not remain in NS-SEC Class 1 following the LGG diagnosis. What could drive this socioeconomic effect in this population? Several quality-of-life measures were more favorable in the group that remained in NS-SEC Class 1 post-LGG diagnosis, but the directionality of this relationship is not clear; the improved quality of life may drive or be the result of changes in occupational status. Notably, attainment of a university-level qualification was significantly associated with maintenance of socioeconomic advantage (NS-SEC Class 1 group membership) following the LGG diagnosis. When considered on its own and without accounting fully for potential confounders—which was not possible to do in a statistically robust way in a study population of this size—this finding does not provide causal evidence for an effect of educational attainment on occupational outcomes following diagnosis of an LGG. However, if such a causal relationship were to exist, this would align with the concept of cognitive reserve, which is defined in terms of individual differences in the processing of tasks that allow some to cope with brain pathology better than others^40^. Cognitive reserve is usually evaluated through proxy measures, particularly educational attainment and NART-estimated premorbid intelligence. Interestingly, there was no association between NART-estimated premorbid intelligence and NS-SEC Class 1 group membership post-LGG diagnosis. On face value, this may appear to contradict the theory of a causal role for cognitive reserve in the maintenance of socioeconomic advantage; however, irrespective of whether such a causal role exists, these observations may instead reflect the possibility that NART scores do not truly reflect pre-morbid intelligence; indeed, the NART evaluates an individual’s ability to pronounce words in a specific way that has been deemed to be ‘correct’ according to society’s construction of what is the ‘right’ and ‘wrong’ way to pronounce a word, and is inherently influenced by socioeconomic and cultural factors^20^. Furthermore, the observed association between university-level qualification attainment and occupational status following LGG diagnosis may not be the result of cognitive reserve but instead be mediated by the presence of a supportive social network. This was not specifically evaluated in the current study and, ultimately, further research is required to fully explore the nature of the relationship between cognitive reserve and occupational outcomes following diagnosis of an LGG.

In a systematic review of 30 studies that either considered return-to-work status (n = 19) or used HRQOL scales incorporating work related aspects (n = 11) in patients with LGG, the majority (61.5–100%) were working at the time of surgery, and postoperative return-to-work rates ranged from 31 to 97.1% (mean 73.1%)^17^. Younger age, better neurological status, having a white-collar occupation, working pre-operatively, being the sole bread-winner, use of awake surgery, and greater extent of resection were associated with increased return-to-work status; female sex, older age, poor neurological status, pre-operative history of work absences, slow lexical access speed, and postoperative seizures were negatively related to return-to-work status^17^. However, these data are limited by a lack of granularity regarding the specific occupational classification and whether occupations (and thus, socioeconomic status classification) changed at the individual patient level.

Among patients with gliomas, returning to work is a particular concern among those with LGG, as the majority of patients in this group are young and in employment at the time of the diagnosis. Factors influencing employment status following diagnosis of an LGG are multifactorial and, in addition to the factors mentioned in the preceding paragraph, are likely to include some aspects which are common across glioma populations, including fatigue and depression^41,42^, seizure risk and associated career and driving-related restrictions, as well as other context-specific factors such as language impairments that could present particular challenges to individuals in client- or customer-facing roles, a lack of possible work adjustments due to the nature of the role, or inadequate support from supervisors or occupational physicians^43^. It is likely that variations exist in illness- and work-related expectations between patients and healthcare professionals as well as employers. Tailored and proactive outreach by relevant professionals regarding work-related issues is necessary, as highlighted by a multicenter interview study of 17 patients with primary brain tumors (predominantly LGGs) and 6 caregivers^44^.

An increased incidence of LGG in those with higher socioeconomic status has been noted in a number of previous studies^45,46^. A commonly mooted potential reason for such an association among medical conditions is ascertainment bias related to socioeconomic status; however, the role of such bias in the incidence of LGG is difficult to justify given that no comprehensive screening programs operate for this tumor type (understandably, given the epidemiology of the condition) and, by their very nature, LGGs tend to present acutely (with seizures, for example) without preceding symptoms. Neither is the increased incidence of LGGs in higher socioeconomic status individuals the result of more socioeconomically advantaged individuals developing the more ‘benign’ types of glioma (i.e., LGG and not high-grade gliomas), because multiple studies indicate the relationship between increased glioma incidence and higher socioeconomic status is observed in both low- and high-grade gliomas, including WHO grade IV glioblastoma^45–50^. It is not clear what drives this relationship, necessitating further study.

## Limitations

There are several limitations to the current study, including the relatively small sample size and the lack of a longitudinal design, particularly relating to the cognitive and quality of life assessments. The study was not sufficiently powered to perform detailed statistical analyses evaluating whether relationships exist between results in specific cognitive tests or domains and socioeconomic status. Recruitment challenges are well-recognized within the field of neuro-oncology^51^. The study was likely subject to selection/recruitment bias due to the nature of the study, requiring travel to hospital and completion of questionnaires and detailed cognitive assessment. For the quality-of-life data, no freely available normative UK data of sufficient quality were identified for comparison to the current study population, and therefore data from the USA were used. Use of these data was limited by the challenges of cross-national comparisons and a lack of adjustment for potential cultural differences, and serve only as a rudimentary comparison. The personality trait of resilience, and how it may influence socioeconomic status, was not evaluated in the current study, and is worthy of exploration in future studies. Finally, according to several measures, including occupational status, the majority of the study population was socioeconomically advantaged prior to the LGG diagnosis. This observation aligns with the findings of several previous studies demonstrating an increased incidence of LGG in those with higher socioeconomic status, as discussed earlier. However, this overrepresentation still limits the generalizability of our findings to patients with LGG from less advantaged socioeconomic backgrounds, due to potential variability in factors such as upbringing and access to specific social networks as well as opportunities.

## Conclusion

The diagnosis of a WHO grade II diffuse low-grade glioma is devastating for a patient and their loved ones. Patients who receive this diagnosis, most of whom are relatively young adults in their fourth and fifth decades of life or younger, face many challenges; not least the treatments, many of which have formidable side-effects, and the knowledge that their tumor will progress to a higher-grade glioma over an undefinable period of time. The work presented here has shown the deleterious socioeconomic effects of an LGG diagnosis and indicates a potential protective role of educational attainment (degree status) on occupational status. The association between educational attainment and occupational outcomes following LGG diagnosis may reflect influences of cognitive reserve and/or supportive social networks, but causality has not been proven and further study is required. Associations between quality-of-life measures and occupational status following LGG diagnosis were also identified. The findings of this study support the need for early tailored and proactive outreach by relevant professionals regarding work-related issues for patients with LGG.

## Electronic supplementary material

Below is the link to the electronic supplementary material.

Supplementary Material 1

## Data Availability

The data generated from this study are available from the corresponding author on reasonable request.
