# Supplementary material for: The deleterious effect of WHO grade II diffuse glioma on socioeconomic status as evaluated through occupation
Source: Sci Rep. 2025 Jul 2;15:23426. doi: 10.1038/s41598-025-08240-7 (PMC12222844; doi:10.1038/s41598-025-08240-7)
Supplement: Supplementary file 1 — Supplementary Material 1 [file 41598_2025_8240_MOESM1_ESM.pdf]

Supplementary Information

Title: The deleterious effect of WHO grade II diffuse glioma on socioeconomic status as evaluated through occupation

Authors: Matthew A. Kirkman, MBBS, PhD,[1*] Michael S. C. Thomas, PhD,[2] Andrew Tolmie, PhD[1]

Affiliations:

[1] Department of Psychology and Human Development, UCL Institute of Education, University College London, London, UK

[2] Department of Psychological Sciences, Birkbeck, University of London, London, UK

*Corresponding author:

Dr. Matthew A. Kirkman, MBBS, PhD

Department of Psychology and Human Development

UCL Institute of Education

University College London

25 Woburn Square

London WC1H 0AA

UK

Email: matthew.kirkman.17@ucl.ac.uk

SUPPLEMENTARY METHODS

Indices of Multiple Deprivation

Indices of Multiple Deprivation (IMD) data are based on a relative rank of each small area in England from the most to least deprived. There are 32,844 small areas (referred to as Lower-layer Super Output Areas) in England, each with an average population of 1,500. The IMD 2019 comprises of seven domains, which contribute with differing proportions to the overall index score as follows: income (contributing 22.5%), employment (22.5%), education (13.5%), health (13.5%), crime (9.3%), barriers to housing and services (9.3%), and living environment (9.3%). There are two supplementary income indices, the Income Deprivation Affecting Children Index (IDACI; a measure of the proportion of all children aged 0–15 years living in income deprived families) and Income Deprivation Affecting Older People (IDAOPI; a measure of the proportion of those aged 60 years and over who experience income deprivation). Of note, there is no definite threshold over which an area is classified as deprived, and the IMD represents a *relative* rather than *absolute* measure of deprivation.

The postcode of the house where the participant spent most of their childhood was used to determine the Lower-layer Super Output Area and the relative deprivation of the area where they lived for the majority of their childhood; information on the relative deprivation was generated for all of the above-listed domains and an overall index score was also generated. As the data only cover postcodes in England, it was not possible to calculate the IMD for participants who spent most of their childhood outside of England.

Scoring of the cognitive tests

Mini-Mental State Examination

The MMSE [1] is a validated and widely used screening measure of cognitive function. It covers the domains of orientation to time and place, attention and calculation (through spelling ‘WORLD’

backwards and the serial subtraction of 7 starting from 100, respectively), language (naming, repetition, comprehension, reading, writing, and copying), and immediate and delayed recall (of three words).

The standard scoring technique for the MMSE is to simply summate the total number of correct answers. An MMSE score was generated for each participant using this approach, generating a non-negative integer with a maximum possible value of 30. Higher MMSE scores indicate better cognitive function and, although the cut-off for 'normal' cognition has been variously defined, scores of 24 or 25 and above are often considered normal [2].

Hopkins Verbal Learning Test-Revised (HVLT-R)

The HVLT-R [3] incorporates a list of 12 nouns derived from 3 semantic categories; there are six alternate forms, and each form comprises a different semantic category. The test comprises of three learning trials that measure immediate recall, a delayed recall trial that is administered without forewarning the participant after a delay of 20–25 minutes, and a yes/no delayed recognition trial. The delayed recognition trial comprises a randomized list of 12 target and 12 nontarget words (distractors), six of which are derived from the same semantic categories as the target words (semantically related distractors) and the other six are not (semantically unrelated distractors). Form 4 of the HVLT-R was used in the current study, which focused on birds, articles of clothing, and carpenter's tools.

The final score for each participant was generated using standard measures, as follows. First, the total number of words recalled in the three learning trials and the delayed recall trial were summated (each with a maximum score of 12). The recall scores were also combined to form three additional measures of learning and memory: (1) the total recall score, which was the sum of learning trials 1–3; (2) the

percentage retention score, which was the trial 4 recall divided by the best score in trials 2 and 3 (multiplied by 100); and (3) the recognition discrimination index, which was calculated as the number of true positives minus the number of false positives. The raw scores for four HVLT-R measures, the three measures listed in the preceding sentence as well as the delayed recall score, were converted to T scores using age-based tables provided in the test manuals [4,5]. For the purposes of the current study, the four resulting T scores were summated for each patient and the resulting single HVLT-R score was used in the subsequent analyses. A higher HVLT-R score indicated better performance.

Controlled Oral Word Association Test (COWAT)

The COWAT [6] requires the participant to produce orally as many words as possible beginning with a specified letter during three one-minute trials, with each trial utilizing a different letter. Different three-letter combinations have been used when administering this test, and letter choice is likely to influence the results to an extent due to differences in letter difficulty and word frequency for each letter [7]; the letters C, F, and L were used for word generation when administering the COWAT in the current study.

The final COWAT score was derived by the sum of all acceptable words provided for the three letters tested, corrected for age and educational level using the data in Supplementary Table S1, below (adapted from [8]). A higher COWAT score indicated better performance.

Supplementary Table S1: Score adjustments for the COWAT according to age and years of education

**Age range (years)**	**Education (years)**	**Score adjustment**
25–54	<9	+9
	9–11	+5
	12–15	+3
	16+	-

55–59	<9	+10
	9–11	+7
	12–15	+4
	16+	+1
60–69	<9	+12
	9–11	+9
	12–15	+6
	16+	+3

Table data adapted from [8]

Trail-Making Test (TMT)

The TMT [9] comprises two parts. Part A requires the subject to connect, by making pencil lines, 25 encircled randomly arranged numbers on a paper in numerical order as rapidly as possible. Part B is similar to part A but instead involves 25 encircled numbers and letters that require connections to be made in alternating order (i.e., number, then letter, then number, then letter, and so on). Both parts require perceptual tracking of a sequence and speeded performance, but part B also requires divided attention. In addition to a measure of visual-motor scanning speed (part A) and executive function (part B), the TMT is also used as a measure of visual speed working memory. Thus, the tests are not pure measures.

Scoring for both parts of the TMT was performed based on the time in seconds required to complete each part and, for the purposes of analyses in the current study, the scores for both parts were combined into a single TMT score (i.e., the combined time to complete parts A and B). A higher overall TMT score indicated worse performance.

Stroop Test - Victoria version

There are a number of variations of the Stroop Test available. In the current study, the Victoria version [10] was utilized, which consists of three tasks, each administered using a card containing six rows of four items. In the first task, the participant is presented with a series of 24 dots of four different colors (green, blue, yellow, and red) arranged pseudorandomly in a grid of six lines, so that each of the four colors is presented once in each row and each color is presented six times in total across the task. The participant is instructed to name the colors of the dots as quickly as possible and in order, beginning with the dot in the top left corner and working across the row before moving to the next row. In the second task, the participant is presented with 24 lowercase words presented as four words in each of six rows, and each word is colored in one of the same four colors as in task one. The words (when, hard, and, over) have no relation to the color of the word, and the order of the colors is the same as for task one. In this task, the participant is asked to name the color of the words as quickly as possible, proceeding in the same order as for task one, and to disregard the verbal content of the words. The third task is similar to task two, except the words used are colors (blue, yellow, red, green), but to complicate matters the words are conflicting with the actual color of the word so that the print color never corresponds to the color name; thus, the word ‘blue’ is never shown in blue font, ‘yellow’ never in yellow font, ‘red’ never in red font’, and ‘green’ never in green font. The participant is asked to name the colors in which the words are printed (and not the colors stated by the word) as quickly as possible. The Victoria version of the Stroop Test has a number of advantages over other versions of the Stroop Test. First, it is quick to administer relative to other versions that have more items in each component task (24 in the Victoria version versus 60–112 in others) which, in addition to the efficiency benefits, may also be preferable in the identification of individuals with difficulty completing the tasks. This is because there is evidence that differences in the task performance between younger and older adults in the 100-item version of the Stroop Test are more pronounced in the initial 40 items than the last 60 items, perhaps due to practice effects later in the task [11]. Another advantage is the availability of a reasonable normative database for the Victoria version.

This task evaluates a participant's inhibitory control as it requires them to inhibit an automatic reading response and produce a color-naming response (which is more effortful for the participant). For scoring of the Victoria version of the Stroop Test, the time taken to complete each task, as well as the number of non-self-corrected errors made, were noted. Most analyses of the Stroop Test in the literature focus on an 'interference effect'. Although an interference effect can be evaluated by calculating the extra time required to name colors in the interference task (task three) relative to the time required to name colors in the control task (task one), it has been shown that differences can be influenced by age-related slowing [12,13]; to mitigate this, use of a Ratio Index of Interference is recommended whereby the time taken to complete task three is divided by the time taken to complete task one [12]. This interference ratio, where higher values indicate worse performance, was the variable used for analysis in the current study.

Hayling Sentence Completion Test

The Hayling Test [14] is divided into two sections, each comprising a set of 15 sentences in which the last word is missing. The first part of each sentence is read out by the investigator, with each sentence clearly incomplete, requiring the participant to complete the sentence by responding orally with a word to complete the sentence as quickly as possible. In Section 1 of the Hayling Test, referred to as 'sensible completion', the participant is asked to provide an appropriate (i.e., logical) word to complete the sentence. For example, for the incomplete sentence "The old house will be torn…", an appropriate response from the participant would be "down". In Section 2 of the Hayling Test, referred to as 'unconnected completion', the participant is asked to intentionally complete the sentence with an illogical word that is completely unconnected to the incomplete sentence. For example, for the incomplete sentence "The captain wanted to stay with the sinking…" an appropriately illogical response could be "moon". This section of the Hayling Test therefore requires the participant to inhibit a strongly activated, automatic response prior to the generation of a new, illogical response.

The Hayling Test results were scored following the standard procedures listed in the test documentation, which is now summarized. Scoring of Section 1 of the Hayling Test (‘sensible completion’) resulted in a simple measure of response speed, whereas Section 2 (‘unconnected completion’) resulted in two scores: an error score and response latency. The response latency was recorded in whole seconds and not rounded up; thus, for example, a response time up to 0.99 seconds was scored as 0. A raw score was generated for both sections of the Hayling Test based on summating the rounded response latencies. The rounded response latencies were summated for each section of the Hayling Test separately and converted to two scaled scales using tables provided with the testing materials: for Section 1, possible scores in the scaled score range from 1 (“Impaired”) to 7 (“High average”), whereas for Section 2 they range from 1 (“Impaired”) to 8 (“Good”). For Section 2 of the Hayling Test, additional analyses were performed to generate an additional (third) scaled score; to do this, each of the 15 responses is categorized as either being correct (the word produced is completely unconnected to the sentence; scored as zero points), a category B error (the response is somewhat connected to the meaning of the sentence but does not result in direct sentence completion), or a category A error (the participant completes the sentence in an entirely plausible and logical manner). The total number of category A and B errors were summated separately and used to generate an “A score” and “B score”, respectively. The A and B scores were then combined and transformed to a scaled score ranging from 1 (“Impaired”) to 8 (“Good”) using a table provided on the scoring sheet. The three scaled scores were then converted an overall scaled score (ranging from 1 [“Impaired”] to 10 [“Very superior”]) using another table in the test materials. It is this overall scaled score that was used for further analysis in the current study.

Brixton Spatial Anticipation Test

The Brixton Test [14] comprises a 56-page stimulus booklet, in which each page presents the same layout of 10 circles spread across two rows of five circles each, with each circle numbered from 1 to 10. On each page, one of the circles is filled in blue color, and the position of this filled circle changes from one page to the next. The changes in position of the filled circle result from simple rules that

vary without advance warning to the participant. While showing the participant one page at a time, they are asked to decide where they believe the position of the filled circle will appear on the next page, through the application of patterns/rules identified on previous pages of the booklet. Participants can respond either verbally or by pointing to the position. Scoring is based on summating the total number of errors, with the total score converted to a scaled score (ranging from 1 [“Impaired”] to 10 [“Very superior”]) using a table provided in the test materials. Response latency is not recorded for the Brixton Test, only the accuracy of the response. The response to the first item is excluded from the score due to the nature of the test and that this initial response is a guess without prior data to inform the decision-making process. The overall scaled score was the variable used for further analysis in the current study.

Scoring of the quality-of-life measures

European Organisation for Research and Treatment of Cancer (EORTC) Quality of Life Questionnaires (QLQ): QLQ-C30 and -BN20

The EORTC QLQ-C30 is a 30-item instrument designed to measure quality of life in patients with cancer. It is the main ‘core’ module of the quality-of-life questionnaires devised by the EORTC, and asks responds about their experiences across a range of potential issues faced by patients with cancer, such as mobility, self-care, symptoms, and effects on family life, social activities, and finances. Version 3.0 of the questionnaire was used in the current study. The first 28 questions require the respondent to rate the extent to which a specific issue has affected them on a scale from 1 (“Not at all”) to 4 (“Very much”), with 23 of these focusing on the participant’s experience of the named issues or symptoms in the week preceding the questionnaire. The final two questions are summarizing evaluations that ask the participant to rate their overall health and overall quality of life during the preceding week on a scale ranging from 1 (“Very poor”) to 7 (“Excellent”).

Scoring of the EORTC QLQ-C30 was performed in line with the official scoring manual [15], where the 30 items were grouped into three domains (global health status / quality of life, functional scales, and symptom scales / items) and scales, as shown in Supplementary Table S2, below.

Supplementary Table S2: Domains and scales for scoring responses to the EORTC QLQ-C30

Domain	Number of items within the domain	Item range [a]
Global health status / quality of life	2	6
Functional scales		
- Physical functioning	5	3
- Role functioning	2	3
- Emotional functioning	4	3
- Cognitive functioning	2	3
- Social functioning	2	3
Symptom scales / items		
- Fatigue	3	3
- Nausea and vomiting	2	3
- Pain	2	3
- Dyspnea	1	3
- Insomnia	1	3
- Appetite loss	1	3
- Constipation	1	3
- Diarrhea	1	3
- Financial difficulties	1	3

[a] *Defined as the difference between the maximum and minimum responses possible for individual items.*

Scoring based on [15]

A raw score was calculated for each scale as the mean of the scale's component items, as follows:

Raw score = (Item 1 + Item 2 + … + Item n) / n

Following creation of the raw scores for each scale, the final score for each of the functional scales, symptom scales / items, and global health status / quality of life were calculated through linear transformation of the scores to a 0–100 scale, as follows:

For the **functional scales**:

Final score = $\left\{1 - \frac{(raw\ score - 1)}{item\ range}\right\} \times 100$

For the **symptom scales / items** and **global health status / quality of life**:

Final score = $\left\{\frac{(raw\ score - 1)}{item\ range}\right\} \times 100$

where item range is as defined as per the footnote to Supplementary Table S2, above.

The differences in the final score calculation techniques between the functional scales and the symptom scales / items and global health status / quality of life result in a high final scale score representing a higher response level, and specifically:

- a higher level of functioning in the functional scales,

- high quality of life in the global health status / quality of life domain, but
- a high level of symptomatology / problems in the symptom scales / items.

The EORTC QLQ-C30 is complemented by a range of ‘modules’ that are available for a range of specific pathologies, symptoms, and important quality of life issues affecting patients with cancer, including aspects of care (such as patient satisfaction), and the psychological needs or experiences of patients. The EORTC QLQ-BN20 is one of these modules, and focuses on the quality of life of patients with brain tumors. It consists of 20 items that assess a range of potential concerns and symptoms experienced by patients with brain tumors, including future uncertainty as well as visual, mobility, and communication difficulties. All 20 items relate to the respondent’s experience in the week prior to completion of the questionnaire, and responses to all 20 items are provided using a scale ranging from 1 (“Not at all”) to 4 (“Very much”).

Scoring of the EORTC QLQ-BN20 was performed in line with the literature [16]. The categories of the scales and single items are shown in Supplementary Table S3, below.

Supplementary Table S3: Scales and single items in the EORTC QLQ-BN20

**Scale / single item**	**Number of items within the domain**	**Item range** [a]
- Future uncertainty	4	3
- Visual disorder	3	3
- Motor dysfunction	3	3
- Communication deficit	3	3
- Headaches	1	3
- Seizures	1	3
- Drowsiness	1	3
- Hair loss	1	3

- Itchy skin	1	3
-Weakness of legs	1	3
- Bladder control	1	3

[a] *Defined as the difference between the maximum and minimum responses possible for individual items.*

A raw score was calculated for each scale as the mean of the scale's component items, as follows:

Raw score = (Item 1 + Item 2 + … + Item n) / n

Following creation of the raw scores for each scale, all scales and single items were linearly transformed to a 0–100 scale, with higher scores indicative of more severe symptoms, as follows:

Final score = $\left\{\frac{(raw\ score - 1)}{item\ range}\right\} \times 100$

where item range is as defined as per the footnote to Supplementary Table S3, above.

Functional Assessment of Cancer Therapy - Brain (FACT-Br)

The FACT-Br is a 50-item instrument that evaluates general well-being and brain cancer-specific concerns, with the items grouped into the following categories: physical well-being, social / family well-being, emotional well-being, functional well-being, and additional concerns, with the initial four categories representing general items and the latter being used to generate a brain cancer subscale. Each item represents a statement and respondents are asked to indicate their agreement with the statement as it applies, in the preceding seven days, using a Likert-type scale ranging from 0 ("Not at all") to 4 ("Very much").

The standard scoring process for responses was followed (available on the following website: https://www.facit.org/measures/fact-br). In brief, scores were generated for each item, with reverse scoring where indicated, and overall subscale scores were generated as indicated in Supplementary Table S4, below. Reverse scoring was performed by subtracting the participants response from 4.

Supplementary Table S4: Subscales within the FACT-Br and scoring technique

**Subscale**	**Number of items within the subscale**	**Number of reverse-scored items**	**How the subscale score is generated for each participant**
Physical well-being	7	7	The 7 individual item scores (created by reverse scoring) were summed, multiplied by 7, and then divided by the number of the 7 items answered by the participant to generate a **PWB subscale score**
Social / family well-being	7	0	The 7 individual item scores were summed, multiplied by 7, and then divided by the number of the 7 items answered by the participant to generate a **SWB subscale score**
Emotional well-being	6	5	The 6 individual item scores (five created by reverse scoring) were summed, multiplied by 6, and then divided by the number of the 6 items answered by the participant to generate an **EWB subscale score**
Functional well-being	7	0	The seven individual item scores were summed, multiplied by 7, and then divided by the number of the 7 items answered by the participant to generate a **FWB subscale score**
Brain cancer subscale	23	13	The 23 individual item scores (13 created by reverse scoring) were summed, multiplied by 23, and then divided by the number of the 23 items answered by the participant to generate a **BrC subscale score**

Using the subscales generated as indicated in Supplementary Table S4, above, three standard measures were generated using the methodology listed in the scoring guide for the questionnaire, as follows:

FACT-Br Trial Outcome Index (TOI) = PWB score + FWB score + BrCS score (possible score range: 0–148)

FACT-G total score = PWB score + SWB score + EWB score + FWB score (possible score range: 0–108)

FACT-Br total score = PWB score + SWB score + EWB score + FWB score + BrCS score (possible score range: 0–200)

Higher scores in all of the above measures were indicative of a better quality of life.

National Adult Reading Test (NART)

The NART is a single-word, oral reading test that comprises of 50 words that are all irregular in that they violate grapheme-phoneme correspondence rules (an example is the word ‘chord’). The words are provided on individual cards and displayed to participants one at a time, and the participant is asked to read each word without a response time limit. As the words involved are irregular, intelligent guessing should not provide the correct answer, and instead the test is a measure of previous word knowledge (hence why this is a test of crystallized and not fluid intelligence). Furthermore, as the test utilizes only single words, the stimulus that a participant needs to analyze is not complex, thus minimal demands on current cognitive capacity are made. The test originated from the finding that, in

patients with dementia, oral reading is often preserved despite reading for meaning being commonly impaired. A limitation to the accuracy of using a reading test like the NART would be in a patient with speech difficulties (dysphasia); none of the participants in the current study had speech difficulties, and thus this was not relevant to the current study.

There is a reasonable body of evidence supporting the notion that NART scores primarily index premorbid rather than current (i.e., at the time of administration) intelligence (for example, [17]). Although it has been suggested that the NART may not be a good indicator of premorbid IQ in patients with glioma [18], it has been widely used for this purpose in patients with glioma and other brain tumors [19–22]. The commonest measure of cognitive reserve, years of formal education, has been shown in a number of studies to not predict cognitive outcomes in patients with brain tumors [20,21,23]; this is perhaps because the length of education does not give an indication of the *quality* of the education received, which is probably more important in the evaluation of cognitive reserve. Some have noted that the NART is limited by overestimating premorbid ability in those with very low scores and underestimates ability in those with very high scores [24–26]. However, the mean NART error score in the current study was 15.14 out of a maximum of 50, with a standard deviation of 7.499 and a Shapiro–Wilk test value of 0.966 (p=0.652), indicating a relatively small spread of the data and that the data followed a normal distribution. Given these nuances, the NART and data on years of education were both used as measures of cognitive reserve in the current study.

With permission from the participants, their verbal responses to the NART were recorded electronically and stored in an anonymized format for later scoring. The number of incorrectly pronounced words were summated to provide a ‘total NART errors’ score, in line with the standard scoring of the NART [27], and this score was incorporated into linear regression equations to predict scores in the Wechsler Adult Intelligence Scale version IV (WAIS-IV) following a previously published and validated technique based on a British sample [24], as follows:

$$\text{Predicted WAIS-IV Full Scale IQ} = -0.975 \times \text{NART error score} + 126.41$$

The resulting variable was used in the analyses.

SUPPLEMENTARY RESULTS

Descriptive data

Health and performance (functional) status

Participants were asked to evaluate themselves according to the ECOG Performance Status Scale as shown in the table below. The vast majority of participants (n=16; 76.2%) reported themselves to be grade 0, i.e., fully active and able to carry on all pre-disease performance without restriction. Three (14.3%) reported themselves to be grade 1, restricted in physically strenuous activity but ambulatory and able to carry out work of a light or sedentary nature, and two (9.5%) reported themselves to be grade 2, ambulatory and capable of all self-care but unable to carry out any work activities, up and about more than 50% of waking hours. No participants rated themselves as grade 3 (capable of only limited self-care) or grade 4 (completely disabled).

The majority of participants (n=16; 76.2%) were receiving anti-epileptic drugs at the time of study participation, with most (n=12) of these participants on a single anti-epileptic drug, two on dual anti-epileptic drugs, and one each on three and five anti-epileptic drugs. The commonest anti-epileptic drug prescribed was levetiracetam (n=11), followed by lamotrigine (n=7), clobazam (n=2), pregabalin (n=1), clonazepam (n=1), lacosamide (n=1), and metformin (n=1). None of the study participants were taking corticosteroid therapy at the time of study participation. There were, however, a number of other medications taken by study participants, including: statin therapy (n=1), hormone replacement therapy (n=1), antihypertensives (n=2), vitamin D supplements (n=1), analgesia (n=1), thyroid replacement therapy (n=2), antidepressants (n=2), and a proton pump inhibitor (n=1). One participant (n=1; 4.8%) also took cannabis oil and another (n=1; 4.8%) cocaine.

Most of the participants (n=18; 85.7%) consumed alcohol with a variable frequency, two participants were previous but not current consumers of alcohol (9.5%) and one (4.8%) had never consumed

alcohol. To contrast, only one (4.8%) participant claimed to be a current tobacco smoker and, although 8 (38.1%) were ex-tobacco smokers, 12 (57.1%) had never smoked tobacco. Thirteen (61.9%) participants had one or more comorbidities, the commonest being depression (n=3; 14.3%), anxiety (n=2; 9.5%) and hypertension (n=2; 9.5%); see Supplementary Table S5 for a full list. Due to the numbers of participants overall and within these subgroups, further analyses according to alcohol consumption, smoking status, or by comorbidities were not performed.

Supplementary Table S5: Performance (functional) status and medical history of the study participants

Variable	N (%)
**Functional status (Eastern Cooperative Oncology Group Performance Status Scale)**	
- Grade 0: Fully active, able to carry on all pre-disease performance without restriction	16 (76.2%)
- Grade 1: Restricted in physically strenuous activity but ambulatory and able to carry out work of a light or sedentary nature, e.g., light house work, office work	3 (14.3%)
- Grade 2: Ambulatory and capable of all self-care but unable to carry out any work activities; up and about more than 50% of waking hours	2 (9.5%)
- Grade 3: Capable of only limited self-care; confined to bed or chair more than 50% of waking hours	0
- Grade 4: Completely disabled; cannot carry on any self-care; totally confined to bed or chair	0
**Anti-epileptic drugs at the time of study participation**	**16 (76.2%)**
**Number of anti-epileptics taken by participants**	
- 1	12 (57.1%)
- 2	2 (9.5%)
- 3	1 (4.8%)
- 5	1 (4.8%)
**Type of anti-epileptic drug used**	
- Levetiracetam	11 (52.4%)
- Lamotrigine	7 (33.3%)
- Clobazam	2 (9.5%)
- Pregabalin	1 (4.8%)
- Clonazepam	1 (4.8%)

- Lacosamide	1 (4.8%)
- Metformin	1 (4.8%)
**Corticosteroid therapy at the time of study participation**	**0**
**Other current medications / drugs**	
- Statin therapy	1 (4.8%)
- Hormone replacement therapy	1 (4.8%)
- Antihypertensives	2 (9.5%)
- Vitamin D supplement	1 (4.8%)
- Analgesia	1 (4.8%)
- Cannabis oil	1 (4.8%)
- Cocaine	1 (4.8%)
- Thyroid replacement therapy	2 (9.5%)
- Antidepressant	2 (9.5%)
- Proton pump inhibitor	1 (4.8%)
**Alcohol consumption status**	
- Current drinker	18 (85.7%)
- Ex-drinker	2 (9.5%)
- Never drank alcohol	1 (4.8%)
**Tobacco consumption status**	
- Current smoker	1 (4.8%)
- Ex-smoker	8 (38.1%)
- Never smoked	12 (57.1%)
**Comorbidities / past medical and surgical history**	
- Anxiety	2 (9.5%)
- Breast cancer (ductal carcinoma in situ)	1 (4.8%)
- Chronic fatigue syndrome	1 (4.8%)
- Gallstones (treated with cholecystectomy)	1 (4.8%)
- Hashimoto's disease	1 (4.8%)
- Hypertension	2 (9.5%)
- Hypothyroidism	1 (4.8%)
- Non-epileptic attack disorder	1 (4.8%)
- Gilbert's syndrome	1 (4.8%)
- Osteoarthritis	1 (4.8%)

- Lipoma (excised)	1 (4.8%)
- Bowel adenoma (excised)	1 (4.8%)
- Appendicectomy	1 (4.8%)
- Ollier's disease	1 (4.8%)
- Ischemic stroke	1 (4.8%)
- Depression	3 (14.3%)
- Iron-deficiency anemia	1 (4.8%)
- Sciatica	1 (4.8%)

Tumor characteristics

The tumor characteristics of the study cohort are shown in Supplementary Table S6, below. The majority (n=14; 66.7%) of patients were diagnosed as having a WHO grade II oligodendroglioma, and the remainder of patients were diagnosed as having a WHO grade II diffuse astrocytoma (n=6; 28.6%) or WHO grade II oligoastrocytoma (n=1; 4.8%). Given the changes in the WHO classification of brain tumors over the past few years, the patient diagnosed with a WHO grade II oligoastrocytoma most likely would be diagnosed with a WHO grade II oligodendroglioma using current diagnostic criteria (particularly given the confirmed presence of a 1p19q co-deletion). From the genetics perspective, *IDH* status, 1p19q co-deletion status, and *ATRX* status are shown in the below table. The majority of tumors were right-sided (n=13; 61.9%), with seven patients (33.3%) having a left-sided tumor and one (4.8%) with bilateral tumors. The frontal lobe was the most commonly affected lobe (n=13; 61.9%), followed by the parietal lobe (n=6; 28.6%), temporal lobe (n=4; 19.0%), insular lobe (n=2; 9.5%), and occipital lobe (n=2; 9.5%). More than one lobe was affected by tumor in five (23.8%) participants.

Supplementary Table S6: Tumor characteristics

Variable	N (%)
**Tumor diagnosis**	
- WHO Grade II oligodendroglioma	14 (66.7%)
- WHO Grade II diffuse astrocytoma	6 (28.6%)
- WHO Grade II oligoastrocytoma	1 (4.8%)
**IDH status**	
- Mutation	18 (85.7%)
- Wild type	1 (4.8%)
- Not tested	1 (4.8%)
**1p19q co-deletion**	
- Yes	15 (71.4%)
- No	6 (28.6%)
**ATRX status**	
- Loss of expression (mutation)	5 (23.8%)
- Retained	11 (52.4%)
- Not tested/reported	5 (23.8%)
**Tumor laterality**	
- Left	7 (33.3%)
- Right	13 (61.9%)
- Bilateral	1 (4.8%)
**Involvement of more than one lobe**	**5 (23.8%)**
**Lobe(s) affected**	
- Frontal	13 (61.9%)
- Temporal	4 (19.0%)
- Insular	2 (9.5%)
- Occipital	2 (9.5%)
- Parietal	6 (28.6%)

Symptoms at presentation

As shown in Supplementary Table S7, in the majority of participants (n=15; 71.4%), the initial symptom or sign at the time of presentation to healthcare providers was seizures, followed by headaches (n=4; 19.0%). One participant (4.8%) presented with visual and cognitive impairment, and another with speech disturbance and vertigo.

Supplementary Table S7: Symptoms and signs at presentation

**Symptom/sign**	**N (%)**
- Seizures	15 (71.4%)
- Headaches	4 (19.0%)
- Visual and cognitive impairment	1 (4.8%)
- Speech disturbance and vertigo	1 (4.8%)

Treatment(s) received

Details of the surgical and non-surgical treatments received by participants are shown in Supplementary Table S8, below. All participants underwent surgery to confirm the histopathological diagnosis. Most (n=15; 71.4%) participants underwent one surgical procedure, but six (28.6%) underwent two. Of the 15 participants who underwent a single operation, two (13.3%) underwent biopsy, six (40%) underwent debulking (partial resection) of the tumor (four [66.7%] with the use of intraoperative MRI), and the remaining seven (46.7%) underwent gross total resection of their tumor (three [42.9%] with the use of intraoperative MRI). Of the six participants who underwent two surgeries, one (16.7%) underwent a biopsy followed by gross total resection four years later. Another participant underwent two surgeries in the same year, the latter with the use of intraoperative MRI, where gross total resection was achieved. The remaining four of the six participants that underwent two surgeries underwent debulking (partial resection) of their tumors, two with the use of

intraoperative MRI and two without. Whether the surgery was performed with the patient awake or asleep is shown in Supplementary Table S8, below.

The mean time between surgery and the initial postoperative MRI scan was 0.7 (SD 1.1) days, which was particularly short due to the use of intraoperative MRI in over half of the cases. The mean time between the last surgery performed and participation in the study was 49.2 (SD 23.4) months.

There were four (19.0%) complications following surgery, affecting four different participants. They were: expressive dysphasia, stroke, supplementary motor area syndrome, and facial weakness.

Six (28.6%) participants received chemotherapy, either temozolomide (TMZ; n=3; 14.3%), procarbazine, lomustine, and vincristine (PCV; n=2; 9.5%), or both TMZ and PCV (n=1; 4.8%). The mean time between the end of chemotherapy and participation in the study was 20.8 (10.5) months. Three complications were reported in two participants who underwent chemotherapy: one patient developed thrombocytopenia and febrile neutropenia, and one patient developed thrombocytopenia alone.

Eight (38.1%) patients received radiotherapy for their brain tumor. In all eight cases, RapidArc Intensity Modulated Radiotherapy was delivered. The specific dosage schedules received by participants are shown in the table below. The mean time between the end of radiotherapy and study participation was 47.8 (26.7) months.

Supplementary Table S8: Details of the treatments received for LGG

**Variable**	**N (%) or mean (SD) / median (range)**
**Number of surgeries**	
- One	15 (71.4%)
- Two	6 (28.6%)
**Time between two surgeries (n=6) in months**	**29.2 (18.1) / 30.3 (1.7–49.3)**
**Extent of tumor resection - first surgery (n=21)**	
- Biopsy	3 (14.3%)
- Debulking (partial resection)	11 (52.4%)
- Gross total resection	7 (33.3%)
**Extent of tumor resection - second surgery (n=6)**	
- Debulking (partial resection)	4 (66.7%)
- Gross total resection	2 (33.3%)
**Craniotomy technique - first surgery (non-biopsy cases only; n=19)**	
- Asleep with intraoperative MRI	5 (26.3%)
- Asleep without intraoperative MRI	7 (36.8%)
- Awake with intraoperative MRI	4 (21.1%)
- Awake without intraoperative MRI	3 (15.8%)
**Craniotomy technique - second surgery (n=6)**	
- Asleep with intraoperative MRI	2 (33.3%)
- Asleep without intraoperative MRI	1 (16.7%)
- Awake with intraoperative MRI	2 (33.3%)
- Awake without intraoperative MRI	1 (16.7%)
**Time between surgery and initial post-operative MRI scan in days**	**0.7 (1.1) / 0 (0–4)**
**Time between last surgery and time of study participation in months**	**49.2 (23.4) / 44.2 (19.3–96.9)**
**Complications following surgery**	
- Expressive dysphasia	1 (4.8%)
- Stroke	1 (4.8%)
- Supplementary motor area syndrome	1 (4.8%)
- Facial weakness	1 (4.8%)
**Chemotherapy, n**	**6 (28.6%)**

**Chemotherapy received**	
- PCV	2 (9.5%)
- TMZ	3 (14.3%)
- Both PCV and TMZ	1 (4.8%)
**Time between end of last chemotherapy cycle and time of study participation in months**	**20.8 (10.5) / 20.0 (6.3–32.2)**
**Complications of chemotherapy**	
- Thrombocytopenia	2 (9.5%)
- Febrile neutropenia	1 (4.8%)
**Radiotherapy, n**	**8 (38.1%)**
**Type of radiotherapy delivered**	
- RapidArc Intensity Modulated Radiotherapy	8 (38.1%)
**Radiotherapy dose schedule**	
- 60 Gy in 30 fractions	1 (4.8%)
- 59.4 Gy in 30 fractions	2 (9.5%)
- 54.9 Gy in 33 fractions	1 (4.8%)
- 54.9 Gy in 30 fractions	3 (14.3%)
- 54 Gy in 30 fractions	1 (4.8%)
**Time between end of last radiotherapy fraction and time of study participation in months**	**47.8 (26.7) / 34.8 (19.9–82.1)**

Gy, gray; MRI, magnetic resonance imaging; PCV, procarbazine, lomustine, and vincristine; SD, standard deviation; TMZ, temozolomide

Socioeconomic status

The socioeconomic information provided by participants is summarized in Supplementary Table S9. Almost half (47.6%) of participants attended a non-selective state school, seven (33.3%) attended a selective state school, and four (19.0%) attended an independent or fee-paying school. Four (19.0%) received free school meals. Most (n=17; 81.0%) did not grow up in households receiving income support and the remainder (n=4; 19.0%) did not know the answer to this. One or both parents had a degree qualification among 8 (38.1%) participants; 11 (52.4%) did not and 2 (9.5%) did not know.

Supplementary Table S9: Socioeconomic information on the study participants

Variable	N (%)
**Type of school attended**	
- State school - non-selective	10 (47.6%)
- State school - selective	7 (33.3%)
- Independent or fee-paying school	4 (19.0%)
**Received free school meals**	**4 (19.0%)**
**Parent degree**	
- Yes	8 (38.1%)
- No	11 (52.4%)
- Do not know	2 (9.5%)
**Household received income support**	
- Yes	0
- No	17 (81.0%)
- Do not know	4 (19.0%)

Parental occupations

The parental occupations of the study participants are shown in Supplementary Table S10.

Supplementary Table S10: Parental occupations

Variable	N (%)
**Father's occupation**	
- Chartered surveyor	1 (4.8%)
- Medical doctor	1 (4.8%)
- Academic professor	1 (4.8%)
- Quantity surveyor	1 (4.8%)
- Ground worker	1 (4.8%)
- Self-employed (owned business)	1 (4.8%)
- Businessman	1 (4.8%)

- Teacher	1 (4.8%)
- Police officer	1 (4.8%)
- Night porter	1 (4.8%)
- Accountant	1 (4.8%)
- Engineer	1 (4.8%)
- Statistician	1 (4.8%)
- Airline pilot	1 (4.8%)
- Salesman	1 (4.8%)
- Manager	1 (4.8%)
- Immigration officer	1 (4.8%)
- Foundry supervisor	1 (4.8%)
- Probation officer	1 (4.8%)
- Courier / lorry driver	1 (4.8%)
- Not specified	1 (4.8%)
**Mother's occupation**	
- Secretary	1 (4.8%)
- Midwife	1 (4.8%)
- Civil servant	1 (4.8%)
- Dental nurse	1 (4.8%)
- Health visitor	1 (4.8%)
- Housewife	3 (14.3%)
- Administrator	1 (4.8%)
- Care assistant	1 (4.8%)
- Nurse	2 (9.5%)
- Nursery key worker	1 (4.8%)
- Social policy	1 (4.8%)
- Hairdresser	1 (4.8%)
- Family therapist	1 (4.8%)
- Cleaner	2 (9.5%)
- Health care assistant	1 (4.8%)
- Unemployed	1 (4.8%)
- Not specified	1 (4.8%)

The parental occupations were classified using the three-class NS-SEC as described in the main manuscript text. The NS-SEC classifications for parents of participants in the current study are shown in Supplementary Table S11. As can be seen from the below table, the majority of fathers (n=14; 66.7%) and the largest proportion of mothers (n=7; 33.3%) of the participants were in the NS-SEC Class 1 group based on their occupation.

Supplementary Table S11: Parental occupations grouped by NS-SEC classification

**Variable**	**N (%)**
**Father's occupation**	
- NS-SEC Class 1	14 (66.7%)
- NS-SEC Class 2	2 (9.5%)
- NS-SEC Class 3	4 (19.0%)
- Unknown / Unemployed / Not specified	1 (4.8%)
**Mother's occupation**	
- NS-SEC Class 1	7 (33.3%)
- NS-SEC Class 2	6 (28.6%)
- NS-SEC Class 3	3 (14.3%)
- Unknown / Unemployed / Housewife / Not specified	5 (23.8%)

Participant NS-SEC class prior to the LGG diagnosis relative to the local population

Through 2 x 2 standard chi-squared testing, the expected and observed frequencies of individuals in NS-SEC Class 1 were evaluated based on census data about the make-up of the population of London. These data revealed that 2,324,632 (35.5%) out of the total of 6,549,173 individuals in London registered in the 2011 Census were classified as being NS-SEC Class 1 using the three-class system. Based on this London-wide proportion, the expected frequency of NS-SEC Class 1 individuals in the study sample would be (2,324,632/6,549,173) x 21=7.5, and thus the proportion of participants in this

study within the NS-SEC Class 1 group prior to their LGG diagnosis was significantly higher than that expected by chance.

Supplementary Table S12: Cross-tabulation of observed versus expected frequencies of NS-SEC classes

	**Observed**	**Expected**
**NS-SEC Class 1**	15	7.45
**Other NS-SEC classes**	6	13.55

Observed data represent that of the study population prior to the LGG diagnosis, whereas expected data are based on proportions of NS-SEC classes within the wider London population according to 2011 Census data

Indices of Multiple Deprivation 2019

Supplementary Table S13: Overall IMD, subdomain, and supplementary indices data of study participants

Less deprived

Decile	IMD, n (%)	Income, n (%)	Employment, n (%)	Education, n (%)	Health, n (%)	Crime, n (%)	Barriers to housing and services, n (%)	Living environment, n (%)	IDACI, n (%)	IDAOPI, n (%)
10	2 (14.3%)	5 (35.7%)	5 (35.7%)	3 (21.4%)	5 (35.7%)	1 (7.1%)	1 (7.1%)	2 (14.3%)	3 (21.4%)	3 (21.4%)
9	4 (28.6%)	1 (7.1%)	2 (14.3%)	1 (7.1%)	3 (21.4%)	1 (7.1%)	0	1 (7.1%)	3 (21.4%)	3 (21.4%)
8	1 (7.1%)	0	0	2 (14.3%)	2 (14.3%)	3 (21.4%)	1 (7.1%)	0	0	0
7	0	1 (7.1%)	2 (14.3%)	2 (14.3%)	1 (7.1%)	0	1 (7.1%)	2 (14.3%)	0	0
6	2 (14.3%)	2 (14.3%)	0	1 (7.1%)	0	3 (21.4%)	1 (7.1%)	2 (14.3%)	3 (21.4%)	2 (14.3%)
5	0	0	3 (21.4%)	3 (21.4%)	1 (7.1%)	1 (7.1%)	2 (14.3%)	0	0	1 (7.1%)
4	3 (21.4%)	3 (21.4%)	1 (7.1%)	1 (7.1%)	0	1 (7.1%)	3 (21.4%)	1 (7.1%)	2 (14.3%)	1 (7.1%)
3	1 (7.1%)	2 (14.3%)	0	1 (7.1%)	1 (7.1%)	0	1 (7.1%)	3 (21.4%)	2 (14.3%)	3 (21.4%)
2	1 (7.1%)	0	1 (7.1%)	0	1 (7.1%)	1 (7.1%)	2 (14.3%)	2 (14.3%)	1 (7.1%)	0
1	0	0	0	0	0	3 (21.4%)	2 (14.3%)	1 (7.1%)	0	1 (7.1%)
Not available	7	7	7	7	7	7	7	7	7	7

More deprived

IDACI, Income Deprivation Affecting Children Index; IDAOPI, Income Deprivation Affecting Older People Index; IMD, Indices of Multiple Deprivation

Percentages calculated after excluding participants (n=7) where deciles could not be calculated.

Cognitive assessment

Supplementary Table S14: Cognitive test results

Test name	Domain(s) evaluated	Outcome measures	Scores, N (%) or mean (SD) / median (range)
MMSE	Global cognitive function	Number of correct answers<br>Maximum score possible: 30	29.0 (1.1) / 29 (27–30)
HVLT-R	Memory	Two parts - part A (free recall) and part B (recognition). Total number of words correctly recalled during immediate recall (HVLT-R Total Recall, trials 1-3); total number of words correctly identified after a delay (HVLT-R Delayed Recall, trial 4); Percentage retention, calculated by dividing HVLT-R Delayed Recall score by the best score from Trials 2 and 3, multiplied by 100 (HVLT-R % Retention); and a recognition discrimination index calculated by subtracting the number of false positives from the number of true positives obtained during part B of the HVLT-R (HVLT-R Recognition Discrimination Index). An age-adjusted T-score is generated for each of these, which is then summated along with individual T scores for Trials 1–3 to generate an overall HVLT-R T score.	Raw scores:<br>HVLT-R Trial 1: 6.2 (2.2) / 6 (3–12)<br>HVLT-R Trial 2: 8.6 (1.8) / 9 (5–12)<br>HVLT-R Trial 3: 9.7 (1.8) / 10 (5–12)<br>HVLT-R Total Recall: 24.6 (4.1) / 25 (14–32)<br>HVLT-R Delayed Recall: 8.2 (2.3) / 8 (3–11)<br>HVLT-R % Retention: 81.7% (19.9) / 83.3% (25–125%)<br>HVLT-R Recognition Discrimination Index: 10.1 (1.8) / 10 (6–12)<br><br>T scores:<br>HVLT-R Trial 1: 42.7 (12.2) / 41 (25–75)<br>HVLT-R Trial 2: 43.5 (11.5) / 44 (20–65)<br>HVLT-R Trial 3: 43.1 (12.2) / 44 (20–59)<br>HVLT-R Total Recall: 42.1 (9.9) / 44 (20–61)<br>HVLT-R Delayed Recall: 41.3 (10.8) / 41 (20–55)

			HVLT-R % Retention:43.1 (12.4) / 44 (20–76)<br>HVLT-R Recognition Discrimination Index: 44.3 (12.2) / 41 (20–60)<br>HVLT-R Overall T score: 300.1 (56.1) / 290 (166–398)
COWAT	Verbal fluency	Number of words correct (with score adjustment for age and years of education)	43.2 (11.1) / 42 (21–62) (post-adjustment scores)
TMT	Part A: Visual-motor scanning speed<br>Part B: Executive function<br>Both: Visual speed working memory	Time to complete tests	Part A: 32.6 s (16.4) / 26 s (17–72 s)<br>Part B: 86.3 s (39.6) / 71 s (50–178 s)<br>Combined A + B: 118.9 s (52.6) / 99 s (74–196 s)
Stroop Test (Victoria version)	Inhibitory control	Time to complete tasks, number of errors, Stroop difference score (time to complete colors task - time to complete dots task), and Stroop interference ratio (time to complete colors task divided by the time to complete the dots task)	Time to complete task<br>Dots: 12.6 s (2.7) / 12 s (6–18)<br>Words: 15.1 s (3.9) / 15 s (7–24)<br>Colors: 24.2 s (7.6) / 22 s (13–39)<br>Number of errors<br>Dots: 0 (0) / 0 (0)<br>Words: 0 (0) / 0 (0)<br>Colors: 0.2 (0.5) / 0 (0–2)<br>Stroop difference score

			11.6 s (5.9) / 10 s (5–25)<br>Stroop interference ratio<br>1.9 (0.4) / 1.9 (1.4–2.9)
Hayling Sentence Completion Test	Response initiation / suppression	Time to complete sentences and classification of errors according to extent of connectedness to sentence. Final measure generated: Hayling Overall Scaled Score (1–10)	Hayling Overall Scaled Score, n (%):<br>10 (very superior) = 0<br>9 (superior) = 0<br>8 (good) = 1 (4.8%)<br>7 (high average) = 1 (4.8%)<br>6 (average) = 16 (76.2%)<br>5 (moderate average) = 2 (9.5%)<br>4 (low average) = 1 (4.8%)<br>3 (poor) = 0<br>2 (abnormal) = 0<br>1 (impaired) = 0
Brixton Spatial Anticipation Test	Visuospatial sequencing / rule attainment	Total number of errors used to generate a scaled score	Brixton Scaled Score, n (%):<br>10 (very superior) = 1 (4.8%)<br>9 (superior) = 0<br>8 (good) = 1 (4.8%)<br>7 (high average) = 5 (23.8%)<br>6 (average) = 7 (33.3%)<br>5 (moderate average) = 2 (9.5%)

4 (low average) = 1 (4.8%)

3 (poor) = 1 (4.8%)

2 (abnormal) = 2 (9.5%)

1 (impaired) = 1 (4.8%)

COWAT, Controlled Oral Word Association Test; HVLT-R, Hopkins Verbal Learning Test - Revised; MMSE, Mini-Mental State Examination; SD, standard deviation; TMT-A, Trail Making Test Part A; TMT-B, Trail Making Test Part B

Quality of life

Supplementary Table S15: A comparison of FACT-G results between the current study and normative data

Domain	Responses from the current study	General adult US population norms	General cancer patient norms
PWB score	22.86 (3.66)	22.7 (5.4)	21.3 (6.0)
SWB score	22.37 (4.67)	19.1 (6.8)	22.1 (5.3)
EWB score	17.43 (4.04)	19.9 (4.8)	18.7 (4.5)
FWB score	21.10 (4.85)	18.5 (6.8)	18.9 (6.8)
FACT-G total score	83.75 (13.73)	80.1 (18.1)	80.9 (17.0)

Data are presented as mean values (standard deviation). PWB, physical well-being; SWB, social well-being; EWB, emotional well-being; FWB, functional well-being. Normative data source: [28]. The general adult US population data were sourced from a random sample of 1,400 people aged 18 years or older selected from over 100,000 members of an internet-based survey panel. The final group comprised of 1,075 individuals aged 18–91 years declared to be a demographically representative sample of the general US adult population. The general cancer patient data were sourced from 2,236 patients originating from two sources: (1) a 3-year validation study of the FACT measurement system, which collected data from patients located in public and private care settings in Atlanta, GA, Chicago, IL, and San Juan, Puerto Rico. Patients were required to be older than 17 years and diagnosed with breast cancer, lung cancer, colorectal cancer, head and/or neck cancer, and/or HIV-related malignancy. Participants were consecutively recruited with the goal of obtaining a reasonably representative patient sample regarding performance status, socioeconomic status, age, and gender; and (2) a different 3-year study that aimed to co-calibrate five health-related quality of life questionnaires, with data collected from patients with cancer in Philadelphia, PA, Baltimore, MD, Toledo, OH, and Chicago, IL. Patients were required to be older than 17 years and have a diagnosis of any cancer or HIV, be at least 2 months post-diagnosis and have a life expectancy of at least 3 months.

REFERENCES

1. Folstein MF, Folstein SE, McHugh PR. “Mini-mental state”. A practical method for grading the cognitive state of patients for the clinician. J Psychiatr Res. 1975;12:189–98.

2. Salis F, Mandas A, as. Cognitive Assessment with Neurocognitive Screening Tools. Journal of Aging Science. 2021;9:1000001.

3. Benedict RHB, Schretlen D, Groninger L, Brandt J. Hopkins Verbal Learning Test – Revised: Normative Data and Analysis of Inter-Form and Test-Retest Reliability. The Clinical Neuropsychologist. 1998;12:43–55.

4. Benedict RHB, Brandt J. Hopkins Verbal Learning Test-Revised/Brief Visuospatial Memory Test-Revised: Professional Manual Supplement. Lutz, FL: PAR; 2001.

5. Brandt J, Benedict RHB. Hopkins Verbal Learning Test-Revised: Professional Manual. Lutz, FL: PAR; 2001.

6. Benton A, Hamsher K. Multilingual aphasia examination. Second. Iowa City: AJA Associates; 1989.

7. Borkowski JG, Benton AL, Spreen O. Word fluency and brain damage. Neuropsychologia. 1967;5:135–40.

8. Benton A, Hamsher K, Rey G, Sivan A. Multilingual aphasia examination. 3rd edition. Iowa City: AJA Associates; 1994.

9. Lezak M, Howieson D, Loring D. Neuropsychological assessment. 4th ed. Oxford New York: Oxford University Press; 2004.

10. Regard M. Cognitive Rigidity and Flexibility [microform] : a Neuropsychological Study. University of Victoria; 1981.

11. Klein M, Ponds RW, Houx PJ, Jolles J. Effect of test duration on age-related differences in Stroop interference. J Clin Exp Neuropsychol. 1997;19:77–82.

12. Graf P, Uttl B, Tuokko H. Color- and picture-word Stroop tests: performance changes in old age. J Clin Exp Neuropsychol. 1995;17:390–415.

13. Verhaeghen P, De Meersman L. Aging and the Stroop effect: a meta-analysis. Psychol Aging. 1998;13:120–6.

14. Burgess P, Shallice T. The Hayling and Brixton Tests. Bury St Edmunds: Thames Valley Test Company; 1997.

15. Fayers P, Aaronson N, Bjordal K, Groenvold M, Curran D, Bottomley A, et al. The EORTC QLQ-C30 Scoring Manual (3rd Edition). Brussels: European Organisation for Research and Treatment of Cancer; 2001.

16. Taphoorn MJB, Claassens L, Aaronson NK, Coens C, Mauer M, Osoba D, et al. An international validation study of the EORTC brain cancer module (EORTC QLQ-BN20) for assessing health-related quality of life and symptoms in brain cancer patients. Eur J Cancer. 2010;46:1033–40.

17. Crawford JR, Deary IJ, Starr J, Whalley LJ. The NART as an index of prior intellectual functioning: a retrospective validity study covering a 66-year interval. Psychol Med. 2001;31:451–8.

18. Ebmeier KP, Booker K, Cull A, Gregor A, Goodwin GM, O'carroll RE. The validity of the national adult reading test in estimating premorbid intellectual ability in long-term survivors of hemispheric glioma and whole brain irradiation—a pilot study. Psycho-Oncology. 1993;2:133–7.

19. Awwad S, Cull A, Gregor A. Long-term survival in adult hemispheric glioma: Prognostic factors and quality of outcome. Clinical Oncology. 1990;2:343–6.

20. Campanella F, Arcara G, Crescentini C, Fabbro F, Skrap M. Cognitive reserve protects language functions in patients with brain tumours. Neuropsychologia. 2021;154:107769.

21. MacPherson SE, Healy C, Allerhand M, Spanò B, Tudor-Sfetea C, White M, et al. Cognitive reserve and cognitive performance of patients with focal frontal lesions. Neuropsychologia. 2017;96:19–28.

22. MacPherson SE, Allerhand M, Gharooni S, Smirni D, Shallice T, Chan E, et al. Cognitive Reserve Proxies Do Not Differentially Account for Cognitive Performance in Patients with Focal Frontal and Non-Frontal Lesions. J Int Neuropsychol Soc. 2020;26:739–48.

23. Kaleita TA, Wellisch DK, Cloughesy TF, Ford JM, Freeman D, Belin TR, et al. Prediction of Neurocognitive Outcome in Adult Brain Tumor Patients. J Neurooncol. 2004;67:245–53.

24. Bright P, Hale E, Gooch VJ, Myhill T, van der Linde I. The National Adult Reading Test: restandardisation against the Wechsler Adult Intelligence Scale-Fourth edition. Neuropsychol Rehabil. 2018;28:1019–27.

25. Mathias JL, Bowden SC, Barrett-Woodbridge M. Accuracy of the Wechsler Test of Adult Reading (WTAR) and National Adult Reading Test (NART) when estimating IQ in a healthy Australian sample. Australian Psychologist. 2007;42:49–56.

26. Nelson H, Wilson J. National Adult Reading Test (NART). Windsor: NFER-Nelson; 1991.

27. Nelson HE. National adult reading test (NART): for the assessment of premorbid intelligence in patients with dementia ; test manual. Windsor: NFER-Nelson; 1982.

28. Brucker PS, Yost K, Cashy J, Webster K, Cella D. General population and cancer patient norms for the Functional Assessment of Cancer Therapy-General (FACT-G). Eval Health Prof. 2005;28:192–211.
